# Low risk of some common cancers in women with anorexia nervosa: Evidence from a national record‐linkage study

**DOI:** 10.1111/acps.13566

**Published:** 2023-05-16

**Authors:** Olena Seminog, Dixa B. Thakrar, Anthony C. James, Michael J. Goldacre

**Affiliations:** ^1^ Big Data Institute, Nuffield Department of Population Health University of Oxford Oxford UK; ^2^ Cancer Epidemiology Unit, Nuffield Department of Population Health University of Oxford Oxford UK; ^3^ Department of Psychiatry University of Oxford Oxford UK

**Keywords:** anorexia nervosa, caloric restriction, cancer, epidemiology, risk

## Abstract

**Background:**

Some studies report that women with anorexia nervosa (AN) have lower risk than others of breast cancer, but increased risk of cancers of other sites. No work has been done to quantify the risk in the English population.

**Methods:**

Retrospective cohort study using a national linked dataset of Hospital Episode Statistics for 1999–2021. We selected individuals with a hospital admission for AN, and compared their relative risk (RR) of developing site‐specific cancers, with that in a reference cohort.

**Results:**

We identified 75 cancers in 15,029 women hospitalised with AN. There was a low RR of all cancers combined at 0.75 (95%CI 0.59–0.94), and, notably, low RR for breast cancer 0.43 (0.20–0.81), cancers of secondary and ill‐defined sites 0.52 (0.26–0.93). The RR for parotid gland cancer was 4.4 (1.4–10.6) within a year of first recorded diagnosis of AN. In men, we found 12 cancers in 1413 individuals hospitalised with AN, but no increased risks beyond the first year of diagnosis of AN.

**Conclusions:**

This is the first report on the association between AN and cancers in the all‐England population. The study showed low rates of breast cancer, and of all cancers combined, in women hospitalised with AN. It is possible that some of the metabolic or hormonal changes observed in AN could work as a protective factor for breast cancer. More experimental work is needed to identify and explain these factors. The new finding on the higher risk of salivary gland tumours could inform clinicians caring for patients with AN.

**Highlights**

- A 20‐year follow‐up study of women and men hospitalised with anorexia nervosa in England found no increased risk of all cancers.
- The risk of breast cancer was 57% lower in women with a history of anorexia nervosa when compared to the reference cohort.
- We found a lower risk of ill‐defined, secondary and unspecified malignancies with a rate ratio 0.52 (95%CI 0.26–0.93) and of cervical cancer, rate ratio 0.27 (0.03–0.98).
- The risk of malignant neoplasms of the parotid gland and other salivary glands was 4.4‐times higher in women and increased in men, but the increase was no longer significant after the first year.
- Women with a history of anorexia nervosa have low rates of most common cancers when compared to the reference cohort. This finding did not apply to men.

## 1 INTRODUCTION

Anorexia nervosa (AN) is a mental health disease that affects mainly women in early adolescence with an incidence of 25.66 per 100,000 women in the UK and Ireland.^1^

Although there are currently differing transdiagnostic perspectives, the DSM5 criteria for anorexia nervosa include: 1. Restriction of energy intake leading to a significantly low body weight 2. Intense fear of gaining weight or becoming fat. 3. Disturbance of perception of body weight and or shape.^2^, ^3^

Due to the nature of this disease, AN can be used as a model disease in studies looking into the effects of extreme caloric restriction on human health.^4^ A growing body of evidence, from both experimental and clinical studies, suggests a positive role of caloric restriction in cancer prevention.^5^, ^6^, ^7^ However, AN is different from a diet with controlled caloric restriction, as it presents with malnutrition, induced vomiting and abuse of laxatives and other medications. These factors in combination, or on their own, might increase the risk of cancers.

Population‐based observational studies do not show an overall reduction in the risk of cancers associated with severe energy restrictions.^8^, ^9^ However, studies from Nordic countries and America have shown a reduced incidence of breast cancer in women with AN.^4^, ^10^, ^11^, ^12^ This negative association has biological plausibility: the risk reduction might be mediated through reduced levels of well‐established risk factors, such as low deposition of fatty tissues, reduced number of ovulatory cycles and low levels of steroid hormones.^13^, ^14^, ^15^

The objective of this study was to quantify the risk of all cancers combined and site‐specific cancers in individuals with AN using very large datasets of routinely collected electronic medical records in England. We hypothesised that the overall risk of cancer would not be different in women with AN and the reference cohort, used as controls, but the risk of individual cancers might differ between the two cohorts.

## 2 METHODS

### 2.1 Population and dataset

We used a linked English national dataset of hospital admissions (Hospital Episode Statistics (HES)) and mortality statistics from the 1st of January 1999 to 31st of December 2021. HES comprises routinely collected administrative data on all hospital admissions and day cases in all NHS hospitals in England, with brief statistical records for every admission. Patients managed as “day cases” are admitted to hospital, but do not stay overnight. The linked dataset was provided by NHS Digital. All records of hospital admissions for each individual person, and the death record in the event of death, were linked in a time‐sequenced record for each individual. The conduct and reporting of our study followed the Strengthening the Reporting of Observational Studies in Epidemiology (STROBE) and Reporting of Studies Conducted Using Observational Routinely Collected health Data (RECORD) statements.^16^, ^17^ The investigators had unrestricted access to the dataset.

### 2.2 Patient and public involvement

The investigation did not conduct any interaction or intervention with participants on whom data were obtained. Patients and the public were not involved in the design, analysis, or interpretation of this study. The analysis was done on anonymised data, and therefore we are not able to consult with or disseminate our findings to participants.

### 2.3 Construction of cohorts

The “exposure” cohort of people with AN was constructed by identifying the first recorded hospital admission or day case for AN in individuals under 35 years old in the linked dataset. We put an upper age threshold for the exposure condition only, but not for the outcomes. We did a sensitivity analysis with higher upper age limits for selected cancers where there was some evidence from the literature on positive associations in older people.

We defined AN as the International Classification of Diseases (ICD) Tenth Revision, code F50.0 for Anorexia Nervosa or F50.1 for Atypical Anorexia Nervosa, or ninth revision code 307.1 for Anorexia Nervosa. Of the total AN cohort, 924 (6.1%) women had a diagnosis of Atypical Anorexia Nervosa.

The reference cohort, for comparison with the exposure cohort, consisted of people hospitalised with a range of mainly minor medical conditions, a range of surgical procedures and a range of injuries. Conditions used in the reference cohort, with the Office of Population, Censuses and Surveys (OPCS) code edition 4 for operations and the ICD‐10 code for diagnosis (with equivalent codes used for other coding editions): appendectomy (OPCS4 H01–H03), adenoidectomy (E20), dilation and curettage (Q10–Q11), hip replacement (W37–W39), knee replacement (W40–W42), squint (ICD‐10 H49–H51), cataract (H25), otitis (H60–H67), upper respiratory tract infections (J00–J06), varicose veins (I83), haemorrhoids (I84), deflected septum, nasal polyp (J33 + J34.2), impacted tooth and other disorders of teeth (K00–K03), inguinal hernia (K40), head injury (S06), in‐growing nail, toenail and other diseases of nail (L60), contraceptive management (Z30), internal derangement of knee (M23), bunion (727.1), dislocations, sprains and strains (S03, S13, S23, S33, S43, S53, S63, S73, S83, S93), selected limb fractures (S42, S52, S62, S82, S92), superficial injury and contusion (S00, S10, S20, S30, S40, S50, S60, S70, S80, S90). From analyses of colorectal cancers, we excluded appendectomy, haemorrhoids and inguinal hernia from the reference cohort. From analysis of uterine cancer we excluded dilation and curettage. These conditions and operations, both individually and in combination, were selected as conditions that were considered very unlikely to be associated with either an atypically high or low risk of cancers.^18^

We have experience in using the reference cohort in other studies of AN and studies of cancers, and have shown that they do not produce atypical values.^19^ Individuals in the exposure cohort and the reference cohort were stratified by age (in 5 year groups), calendar year of first hospitalisation, region of residence, and social class as measured by the Index of Multiple Deprivation. All calculations of the expected and observed number of cancers (see below) were undertaken within these strata (i.e. they were based on people who were the same, in respect of age group, social class, etc) and were then summed across strata to give overall expected and observed number of cases for each cancer. We included all people eligible to be in the reference cohort: the risk of breast cancer in 1378 women aged 25–29 in the AN cohort was compared with the risk in the 130,791 (1378 x 95) women in the reference cohort in the same age group. The ample numbers in the reference cohort allowed us to match one individual in the AN cohort to multiple individuals in the reference cohort.

The individuals in the exposure and reference cohorts were followed for any subsequent hospital admission for or death from each cancer. We used the ICD 9 and ICD 10 codes at three digits for the site‐specific cancers. We grouped some less common forms of cancers into broader groups, see Table 2. Each patient with AN could have contributed to the analysis more than once if they had subsequent cancers of different sites.

In each analysis of each cancer, we excluded individuals with a record of the cancer before their first recorded admission for AN, and those with both conditions first recorded on the same hospital admission. This was done to avoid surveillance bias, since cancer could have been detected as a result of an initial admission for AN, as well as to minimise reverse causality, since anorexia or cachexia might be a sign of cancer.

The hospital admission for AN might increase chances of being diagnosed with other conditions as a result of being under medical care and attending the follow‐up appointments. Hence, we calculated rates in three time intervals, overall, in the first year after hospital admission for AN, and a year or more after first hospital admission for AN.

Separate analysis was done for men hospitalised with AN. However, the numbers were small. We complied with the data protection agreement, which precludes the publication of results with fewer than five observed cases. Therefore, only statistically significant findings of rate ratios without the number of events are reported here.

### 2.4 Statistical analysis

The rates were calculated based on person‐years‐at‐risk. Separate analyses were done for each cancer as described here, using the example of breast cancer. The “date of entry” into the AN or reference cohort, was the date of first admission for AN, or the reference condition. The “date of exit” was the date of subsequent admission for breast cancer, death, or the end of the dataset (31 December 2021), whichever was the earliest. Individuals were censored from further follow‐up on the exact date of first admission for breast cancer or death.

The rate ratio (RR) was standardised using the indirect method, taking the combined AN cohort and reference cohort as the standard population. The “expected” number of cancers in each cohort was calculated by applying the stratum‐specific rates in the standard population, first to the AN cohort, and then, separately, to the reference cohort. The ratio of the standardised rate of occurrence of breast cancer in the AN cohort was calculated relative to that in the reference cohort using the formula (O^AN^/E^AN^)/(O^ref/^E^ref^), where O is the observed and E is the expected number of cases of breast cancer in each cohort. This analysis follows methods for cancer analysis in cohort studies described by Breslow and Day and used by us in other studies of cancers and AN.^20^ The analysis was done using a suite of programmes developed by the Unit of Health‐Care Epidemiology, Oxford, using SAS 9 software (SAS Institute, Cary, NC, USA). We performed Bonferonni corrections for multiple comparisons.

## 3 RESULTS

There were 15,029 women under 35 years old hospitalised with AN contributing 127,184 person‐years at risk. The mean follow‐up in the AN cohort was 9.4 years and the mean age was 18.4 years. The age distribution of the women with AN is provided in Table 1. The reference cohort comprised 1,526,298 women, stratified by the same age groups as the women with AN.

**TABLE 1:** Age distribution of women hospitalised with anorexia nervosa and the reference cohort, 1999–2021, England

Age	Women with AN		Reference cohort	
	*N* (% of total)	Person‐years at risk	*N* (% of total)	Person‐years at risk
<15	3776 (25.1%)	30,375	963,006 (63%)	11,181,868
15–19	6480 (43.1%)	52,864	150,459 (9.9%)	1,514,605
20–24	2433 (16.2%)	21,146	139,646 (9.1%)	1,443,869
25–29	1378 (9.2%)	12,864	130,791 (8.6%)	1,596,785
30–34	962 (6.4%)	9935	142,396 (9.3%)	2,164,146
All <35	15,029 (100.0%)	127,184	1,526,298 (100.0%)	17,901,273

In total, in the analysis of all cancer sites combined, we identified 75 malignant neoplasms, compared to 99.6 expected. The rate ratio with 95% confidence interval, and number of observed and expected cases for cancers overall and for site‐specific cancers are provided in Table 2.

**TABLE 2:** Rate ratio of site‐specific cancers in women with Anorexia Nervosa, with corresponding numbers of observed and expected cases in two time intervals, overall risk, and risk excluding the first year following hospital admission, 1999–2021

Cancer site and ICD10 code	Overall numbers (*N*) and rate ratio (RR)				Rate ratio after the first year following hospital admission was excluded			
	Obs *N*	Exp *N*	RR (95% CI)	*p*‐value	Obs *N*	Expected *N*	(RR 95% CI)	*p*‐value
All cancers (C00‐C75, C81‐C97)	75	99.6	0.75 (0.59–0.94)	0.0152	60	90.0	0.67 (0.51–0.86)	0.0018
All cancers, excluding breast cancer, cervical cancer and ill defined malignancies (C00‐C49, C51‐C52, C54‐C75,C81‐C97)	63	73.4	0.86 (0.66–1.1)	0.0.2453	48	64.8.3	0.74 (0.54–0.98)	0.0421
Breast cancer (C50)	9	21	0.43 (0.20–0.81)	0.01	9	20.2	0.44 (0.20–0.84)	0.0170
Lip, oral cavity and pharynx (C00‐C06)	<5	1.8	1.09 (0.13–3.97)	0.8017	<5	1.5	0.67 (0.02–3.78)	0.9907
Parotid and other salivary glands (C07‐C08)	5	1.2	4.4 (1.4–10.58)	0.002	<5	1	1.93 (0.23–7.16)	0.6554
Oesophagus (C15)	<5	0.5	2.02 (0.05–11.68)	0.9993	<5	0.5	2.04 (0.05–11.81)	0.993
Stomach (C16)	0		N/A		0		N/A	
Small intestine (C17)	<5	0.2	4.09 (0.1–24.36)	0.6147	<5	0.2	4.31 (0.11–25.75)	0.8918
Colon (C18)	<5	3.7	0.54 (0.07–1.96)	0.5332	0	3.4	N/A	
Rectum (C19‐C20)	<5	3.7	0.54 (0.07–1.97)	0.5411	<5	3.4	0.58 (0.07–2.11)	0.6126
Liver (C22)	<5	0.7	1.49 (0.04–8.49)	0.8328	<5	0.6	1.58 (0.04–9.01)	0.8646
Biliary system (C23‐24)	0		N/A		0	0	N/A	
Pancreas (C25)	0		N/A		0		N/A	
Other digestive organs (C26)	<5	0.5	2.22 (0.06–12.91)	0.9458	<5	0.4	2.8 (0.07–16.3)	0.818
Upper respiratory tract (C30‐C33)	<5	0.6	1.65 (0.04–9.46)	0.8867	<5	0.5	1.91 (0.05–11.05)	0.9705
Lung/bronchus (C34)	<5	1.9	1.05 (0.13–3.81)	0.7657	<5	1.8	1.08 (0.13–3.94)	0.7966
Thymus, heart, mediastinum, pleura, other and ill‐defined sites in the respiratory system and intrathoracic (C37‐C39)	0		N/A		0		N/A	
Bone and cartilage (C40‐C41)	<5	2.2	1.35 (0.28–3.98)	0.859	<5	1.7	1.19 (0.14–4.37)	0.8908
Malignant skin melanoma and other malignant neoplasms of skin (C43‐C44)	14	20.1	0.69 (0.38–1.17)	0.2085	13	18.6	0.7 (0.37–1.19)	0.2342
Malignant mesothelioma and soft tissue malignancy (C45‐C49)	<5	3.4	0.59 (0.07–2.15)	0.6311	0		N/A	
Female genital organs, including ovarian cancer (C51‐C52 and C54‐C58)	7	8.3	0.84 (0.34–1.73)	0.7707	4	7.7	0.52 (0.14–1.33)	0.2459
Cervical cancer (C53)	<5	7.4	0.27 (0.03–0.98)	0.0714	<5	7	0.28 (0.03–1.03)	0.0879
Urinary system (C64‐C68)	<5	2.4	1.25 (0.26–3.68)	0.9489	<5	2.3	1.34 (0.27–3.94)	0.8673
Eye, brain and other parts of central nervous system (C69‐C72)	10	6.1	1.66 (0.79–3.06)	0.1621	9	4.9	1.85 (0.84–3.54)	0.8673
Thyroid gland (C73)	5	6.1	1.66 (0.79–3.06)	0.1621	5	5.4	0.92 (0.3–2.16)	0.9722
Adrenal gland and other endocrine glands (C74‐C75)	0		N/A		0		N/A	
Ill‐defined, secondary and unspecified sites (C76‐C80)	15	23	0.65 (0.36–1.07)	0.1165	11	21	0.52 (0.26–0.93)	0.0369
Hodgkin's lymphoma (C81)	<5	3.6	0.5 (0.1–1.47)	0.3074	<5	3.1	0.57 (0.12–1.69)	0.4538
Non‐Hodgkin's lymphoma (C82‐C85)	6	6	0.5 (0.1–1.47)	0.3074	<5	4.7	0.85 (0.23–2.2)	0.9326
Multiple myeloma (C90)	0		N/A		0		N/A	
Acute lymphoblastic leukaemia (C91.0)	0		N/A		0		N/A	
Chronic lymphocytic leukaemia (C91.1)	0		N/A		0		N/A	
Acute myeloid leukaemia (C92.0)	0		N/A		0			
Chronic myeloid leukaemia (C92.1)	0		N/A		0			

The risk of all cancers combined (C00‐C75 and C81‐C97) was low, RR 0.75 (95% confidence interval (CI) 0.59–0.94)) (Table 2). The risk of breast cancer was significantly low, RR 0.43 (0.20–0.81), based on 9 observed and 21 expected cases. We also found a low risk of ill‐defined, secondary and unspecified malignancies (C76‐C80) with RR 0.52 (0.26–0.93) based on 11 observed and 21 expected cases when the first year after hospital admission for AN was excluded. The overall risk of cervical cancer was low, but the reduction became non‐significant after the first year of follow‐up was excluded, perhaps, due to a small number of observed cases. To check if the overall cancer risk reduction was influenced by the negative association with the three types of cancers with low RR, we did a separate analysis excluding them. There was no reduction in overall RR for all cancers, 0.86 (0.66–1.1). However, the risk reduction was marginally significant at a longer follow‐up, 0.74 (0.54–0.98).

There was an increase in the risk of malignant neoplasm of the parotid and other salivary glands 4.4 (1.4–10.6); the risk was high in the first year after hospital admission for AN 29.5 (5.2–114.4), but non‐significant thereafter, 1.9 (0.2–7.2). We did not find any increased or decreased risk of other site‐specific cancers.

We performed separate data analysis for men. There were 12 cancers in 1413 men aged under 35 admitted with AN. The rate ratio for all cancers combined was moderately increased, 2.1 (1.1–3.7), but became non‐significant after the first year following hospital admission was excluded, 1.6 (0.7–3.1). The analysis of site‐specific cancers returned very small numbers of individual cancers, all of them were below five cases. The rate ratios were significantly high for two types of cancer, parotid and other salivary glands 49.1 (5.9–180.4) and rectum 10.6 (2.2–30.9), but only in the first year after hospital admission and non‐significant thereafter.

### 3.1 Sensitivity analysis

#### 3.1.1 Effect of age

Some studies have reported on the increased risk of upper gastrointestinal cancers associated with AN.^21^, ^22^ These studies had a higher age threshold than ours of 35 years old, or no age limit. To check if age has an impact on the risk of oesophageal and stomach cancer, we performed additional analyses increasing the upper age limit. Among women under 45 years, we found that the overall risk of oesophageal cancer was higher 4.5 (1.5–10.7), based on 5 observed and 1.1 expected cases, and after the first year was excluded, the risk was 3.7 (1.0–9.5). No elevated risk of stomach cancer was observed in this age group. In women aged under 55 years, rate of oesophageal cancer was 9.3 (5.6–14.4), based on 20 observed and 2.2 expected cases, and at 1+ years, 6.4 (3.4–10.9), based on 13 observed and 2.1 expected cases. The risk of stomach cancer was 3.1 (1.1–6.8), based on 6 observed and 1.9 expected cases. The risk was significantly high within the first year only, RR 24.70 (4.91–77.04), but was not significant at longer follow‐up, 1.67 (0.34–4.90).

#### 3.1.2 Association with other eating disorders

We did the same analysis as AN for Bulimia Nervosa (BN) and site‐specific cancers, using the ICD‐10th revision codes F50.2 and F50.3. We identified 6091 women admitted with BN. There were no altered rates for any of the cancers in the analysis. The full set of results is provided in Supplementary Table 1.

## 4 DISCUSSION

### 4.1 Principal findings

This is the first study reporting on the association between AN and cancers using routine medical records from England. We found a low rate of occurrence for all cancers combined, in women, but not in men. This reduction was driven by low rates of three cancers, specifically breast cancer, cervical cancer and malignant neoplasms of ill‐defined, secondary and unspecified sites. Once these cancers were removed from the analysis, the overall risk reduction was no longer evident, and the risk no longer differed from the reference population. However, at a longer follow‐up there was a small detectable reduction in risk of all cancers. All reported associations were specific for AN, and no altered RRs were observed in the analysis of BN. We found a high rate of salivary gland cancers associated with AN in women and men, and of oesophageal cancers in older women, notably within a year of first recorded diagnosis with AN.

### 4.2 Comparison with the literature

Whilst there are consistent reports of low risk of breast cancer in individuals with AN, the literature is inconsistent about the overall cancer risk reduction.^4^, ^8^, ^9^, ^10^, ^11^, ^12^ While some reported somewhat lower cancer risk,^7^ others found no significant difference in cancer risk between individuals with AN and the general population.^4^, ^8^, ^23^ In this study we found a modest overall reduction in cancer risk in women with AN. A recent meta‐analysis reported a relative risk of 0.97 (0.89–1.06).^24^ A similar incidence rate ratio, 0.97 (95% CI 0.87–1.08) was reported in the large Scandinavian study based on 366 cases of cancer.^4^ The estimate is very similar to the rate ratio we reported here after excluding the three cancers with low rates. The years covered by the studies included in systematic reviews, as well as the follow‐up duration, overlapped with our study. Hence, the differences between our findings and the literature might be due to differences in populations and specific cancer risks in different countries. There were no studies published using English data to compare our findings to.

We identified individual site‐specific cancers that were driving the risk reduction by systematically assessing the risk associated with each cancer. The lowest relative risk in our AN cohort was for breast cancer at 0.43 (0.2–0.8), which can be translated as 57% risk reduction. The Swedish study reported a similar estimate of 53% reduction in incidence of breast cancer in young women hospitalised with AN.^11^ Similarly, a pooled analysis of data from several countries in Scandinavia reported low rate ratio, at 0.61 (0.49–0.77).^4^ A prospective cohort study from the US also reported a low risk at 0.62 (0.42–0.92).^12^ A recent meta‐analysis and systematic review confirmed the a negative association with breast cancer, reporting a pooled estimate of RR 0.6 (95% CI 0.50–0.80).^24^ Despite the low risk of breast cancer, women with AN who develop breast cancer, have worse survival.^24^

A number of reports found 4 to 6‐times higher risk of upper gastrointestinal cancer in women with AN.^4^, ^21^, ^22^ However, we did not observe an increased risk of oesophageal and stomach cancers in our cohort. This may be due to the differences in age composition between the study cohorts – study from Taiwan had no upper age limit, while the Scottish study had a cut off at 60 years, and the Scandinavian at 50 years. In the additional sensitivity analysis with a higher upper age threshold, we found an increased risk of GI cancers. One mechanism for this might be acid reflux and Barrett's oesophagus in the case of bulimic type AN.^25^ Unfortunately, we did not have a breakdown of restrictive versus bulimic type AN to perform a subgroup analysis. Another important consideration is that it is possible for early GI cancers to present with some clinical features of anorexia nervosa resulting in an initial misdiagnosis.

A Swedish study reported 3 observed cases vs 0.9 expected of ill‐defined and secondary cancers in women with AN and non‐significant Standardised Incidence Ratio 3.2 (95%CI 0.7–9.4).^8^ In our study the total number of cases was not trivial, 15 observed vs 23 expected. This produced non‐significant overall RR 0.65 (0.36–1.07), but the reduction became significant at a longer follow‐up after the cases observed in the first year were excluded, RR 0.52 (0.26–0.93). It is important for future studies to include this ICD10 code range, as well as perform analysis in time‐periods.

#### 4.2.1 Cervical cancer

We found two published studies reporting on the risk of cervical cancer in women with AN, both from the Nordic populations.^4^, ^7^ The findings were consistent with our results, suggesting a somewhat lower risk with a borderline statistical significance. The smaller Danish study had only 2 cases and reported a standardised incidence rate at 0.6 (95% CI 0.1—2.0).^7^ We found fewer than 5 cases and RR was 0.3 (95% CI 0.03–0.98). The larger study with combined data from Sweden, Denmark and Finland, reported 21 cases of cervical cancer with incidence rate ratio 0.7 (0.4–1.0).^4^ We did not detect any risk reduction beyond the first year. However, the Scandinavian study did not report findings in time‐periods. Hence, we do not know if the risk reported in their study remained low after a longer follow‐up.

#### 4.2.2 Salivary gland cancer

There is a literature on salivary gland disorders in individuals with BN and bulimic type of AN.^26^, ^27^, ^28^, ^29^ These patients are known to suffer from sialodenosis, a benign non‐inflammatory enlargement of salivary gland caused by reduction in salivary flow. It usually presents with an asymmetric facial swelling, which might develop acutely after an episode of binge‐purging, or as a long‐term condition in some individuals.^26^, ^27^ A more severe form of a salivary gland injury reported in individuals with BN is a necrotizing sialometaplasia.^29^ This tumour shares some common characteristics with a malignant palatal tumour, and hence might be misdiagnosed as a malignancy. A possibility that it is a misdiagnosis is further supported by our findings that the risk is no longer high at a longer follow‐up. In our dataset, we found an increased risk of salivary gland cancers in AN, but not in BN. Individuals with BN are less likely to have indications for a hospital admission, and perhaps follow a different patient pathway that we do not capture in our hospital dataset.

### 4.3 Strengths and weakness of the study

The use of a nationwide, population‐based dataset of linked electronic patient records from all hospitals, psychiatric and general, in England, provided sufficient numbers and power to study the association between AN and a large number of site‐specific cancers in women aged under 35 years. The unique NHS number assigned to individual patients allowed the linkage of multiple hospital records and any death record belonging to an individual.

Due to the nature of data entered in HES, we did not have information on the type of breast cancer or detailed information about other cancers, or on possible confounding factors (discussed below) associated with both AN and cancers, or on the clinical type of AN. The fact that we did not observe an increased risk of many site‐specific cancers could be explained by the age of individuals in the study. A study with a longer follow‐up of 30–40+ years might provide more information about the delayed consequences of AN in terms of increased cancer risk as suggested by other studies.^22^, ^23^ Another possible, albeit unlikely, explanation for low rates of cancer might be that young women with AN do not seek medical help when they experience symptoms of cancer.

Salivary gland cancer is extremely rare in the general population. Enlarged and hardened parotid glands are a recognised feature of some eating disorders, in particular bulimia or bulimic type AN. It is also possible that such cancers might make eating uncomfortable and, initially, contribute to the possibility of misdiagnosis as AN. We have little information on the clinical characteristics of the patients. Specifically, we do not know whether, once a diagnosis of salivary gland cancer or of oesophageal cancer was made, there was any re‐consideration of the diagnosis of AN; or whether it was judged clinically that the diagnoses of AN and the cancer were appropriate, and that the conditions did indeed co‐exist.

As with any routinely collected data, we did not have information on relevant confounders or mediators. For example, we were not able to ascertain whether women had amenorrhoea or had any information about the age at menarche. Early age at menarche is a risk factor for breast cancer and hence if these women were not menstruating, or started later in life, they may have a lower risk of breast cancer.

It is likely that physiological changes observed in AN patients, whose condition is severe enough to require hospitalisation, would result in low oestrogen plasma levels. These changes secondary to the disturbed hypothalamic–pituitary‐gonadal axis, causing amenorrhoea, could reduce the risk of breast cancer.^30^, ^31^ Unfortunately, we did not have information about the body mass index (BMI) or body composition, at the time of hospital admission or later in life. However, having lower adipose tissue means there is less peripheral conversion of cholesterol to oestrogens, hence the reduction in circulating oestrogens.^32^ In postmenopausal women this mechanism may reduce the risk of breast cancer.^33^

Physical activity was reported to be associated with a reduced risk of breast cancer, with a 20%–40% decrease in risk of breast cancer among the most physically active.^33^ Girls and women with AN are known to exercise excessively. Since we do not have any lifestyle information, we could not stratify our cohort into groups depending on their physical activity levels, and cannot comment on its impact on the risk of cancer. Future studies might be able to address this important limitation.

Hospitalised individuals with AN constitute a well‐defined group of individuals with extremely low oral intake and low weight. These are likely to be more severe cases than those with AN managed in primary care or community services. Therefore, the findings might not be generalizable to all individuals with AN.

### 4.4 Unanswered questions and future research

It is not clear which specific changes in the metabolism, behaviour, body composition, or a combination of these, have led to the observed low rates of all cancers combined, and of breast cancer specifically. We have no information about the BMI at the time of AN admission, or later in life. The relationship between the risk of cancers and body composition and/or BMI should be studied further. Experimental studies are needed to investigate the impact of circulating steroid hormones on breast cancer risk, and changes in the hormone levels caused by caloric restrictions.

It is possible that the risk of breast cancer differs in women with regular and irregular menarche, or/and depending on the total number of ovulatory cycles. Hence, further studies looking at such possible mechanisms are warranted.

Future research on the relationship between AN and cancers should further investigate these site‐specific cancers, and focus on long‐term risks. The longer follow‐up of patients with AN will allow to capture more cancers that typically develop at older age. With the parotid and other salivary gland cancers, we do not know if the eating habits and other behavioural changes associated with AN caused the cancer, or whether the low appetite and anorexia resulted from a subclinical paraneoplastic process that preceded the cancer diagnosis. This would be better studied in a prospective study with a sufficient follow‐up time.

To conclude, this is the first study reporting on the risk of all and site‐specific cancers in AN in the English population. The findings on lower risk of all cancers and specifically breast cancer in women with AN are intriguing. For women recovering from anorexia nervosa, or indeed living with this condition, it is reassuring to know that they are not predisposed to a high risk of cancers in the long run. There is biological plausibility for lower risk of breast cancer reported here. However, the specific mechanisms need to be further investigated in basic research and experimental studies. This publication might prompt researchers to study if energy restriction might play a role in cancer prevention in humans. Our findings on an increased risk of cancers of parotid and other salivary glands should alert clinicians to this problem, in what is already a serious medical condition.

## AUTHOR CONTRIBUTIONS

**Anthony James** proposed the study idea. **Olena Seminog** conceived the study and is the guarantor. **Olena Seminog** did the analysis and wrote the first draft. **Michael J. Goldacre** developed the methodology and designed the study. **Dixa Thakrar** did the literature review. All authors contributed to the interpretation of the data and revised the manuscript. **Olena Seminog** finalised and submitted the manuscript. The corresponding author attests that all listed authors meet authorship criteria and that no others meeting the criteria have been omitted.

## FUNDING INFORMATION

No specific funding was received for this study. OS work was funded by the National Institute for Health Research (NIHR) Oxford Biomedical Research Centre and by Health Data Research UK. MJG is Emeritus Professor. AJ received no funding for this work. DBT is a DPhil student funded by Cancer Research UK. This research was funded in whole, or in part, by the National Institute for Health Research. For the purpose of Open Access, the author has applied a CC BY public copyright license to any Author Accepted Manuscript version arising from this submission.

## CONFLICT OF INTEREST STATEMENT

Authors declare no competing interests.

### PEER REVIEW

The peer review history for this article is available at https://www.webofscience.com/api/gateway/wos/peer-review/10.1111/acps.13566.

## ETHICS APPROVAL

Approval for the programme of work covering the construction and analysis of the linked data set was given by the Central and South Bristol Research Ethics Committee (ref 04/Q2006/176) and has been updated annually. Written informed consent for participation was not required for this study in accordance with the national legislation and the institutional requirements.

## Supporting information


**Supplementary Table 1.** Rate ratio of site‐specific cancers in women with Bulimia Nervosa (BN) with corresponding numbers of observed and expected cases in women hospitalised with BN in two time intervals, overall risk and risk, and risk excluding the first year following hospital admission, 1999–2021.

## Data Availability

The data that support the findings of this study are available from NHS Digital. Restrictions apply to the availability of these data, which were used under license for this study. Data are available from https://digital.nhs.uk/ with the permission of NHS Digital.
