# Supplementary material for: Low risk of some common cancers in women with anorexia nervosa: Evidence from a national record‐linkage study
Source: Acta Psychiatr Scand. 2023 May 16;148(1):71–80. doi: 10.1111/acps.13566 (PMC10953461; doi:10.1111/acps.13566)
Supplement: Supplementary file 1 — Supplementary Table 1. Rate ratio of site‐specific cancers in women with Bulimia Nervosa (BN) with corresponding numbers of observed and expected cases in women hospitalised with BN in two time intervals, overall risk and risk, and risk excluding the first year following hospital admission, 1999–2021. [file ACPS-148-71-s001.docx]

Supplementary table 1. Rate ratio of site-specific cancers in women with Bulimia Nervosa (BN) with corresponding numbers of observed and expected cases in women hospitalised with BN in two time intervals, overall risk and risk, and risk excluding the first year following hospital admission, 1999–2021.

| **Cancer site and ICD10 code** | **Overall rate ratio** | | | |  | **Rate ratio after the first year following hospital admission was excluded** | | | |
| --- | --- | --- | --- | --- | --- | --- | --- | --- | --- |
|  | **Obs N** | **Exp N** | **RR (95%CI)** | **p-value** |  | **Obs N** | **Expected N** | **(RR 95%CI)** | **p-value** |
| All cancers (C00-C75, C81-C97) | 75 | 99.6 | 0.75 (0.59-0.94) | 0.0152 |  | 60 | 90.0 | 0.67 (0.51-0.86) | 0.0018 |
| All cancers, excluding breast cancer (C00-C49, C51-C75,C81-C97) | 60 | 80.2 | 0.81 (0.62-1.03) | 0.0984 |  | 50 | 71.3 | 0.70 (0.52-0.92) | 0.0135 |
| Breast cancer (C50) | 9 | 12.2 | 0.74 (0.34-1.4) | 0.4411 |  | 9 | 11.7 | 0.7 (0.35-1.47) | 0.527 |
| Lip, oral cavity and pharynx (C00-C06) | <5 | 1 | 2.09 (0.25-7.61) | 0.583 |  | <5 | 0.8 | 4.4 (1.4-10.58) | 0.395 |
| Parotid and other salivary glands (C07-C08) | <5 | 0.6 | 1.7 (0.04-9.67) | 0.01 |  | 0 | 0.5 | N/A | N/A |
| Oesophagus (C15) | 0 | 0.3 | N/A |  |  | 0 | 0.3 | N/A |  |
| Stomach (C16) | 0 |  |  |  |  | 0 |  |  |  |
| Small intestine (C17) | 0 | 0.1 | N/A |  |  | 0 | 0.1 | N/A |  |
| Colon (C18) | <5 | 1.9 | 1.08 (0.13-3.92) | 0.07 |  | <2 | 1.7 | 1.17 (0.14-4.25) | 0.870 |
| Rectum (C19-C20) | <5 | 1.9 | 0.52 (0.01-2.89) | 0.753 |  | 0 | 1.8 | N/A |  |
| Liver (C22) | 0 | 0.3 | N/A |  |  | 0 | 0.3 | N/A |  |
| Biliary system (C23-24) | 0 |  | N/A |  |  | 0 | 0 | N/A |  |
| Pancreas (C25) | <5 | 0.4 | 2.75 (0.07-15.68) | 0.824 |  | 0 | 0.3 | N/A |  |
| Other digestive organs (C26) | 0 | 0.2 | N/A |  |  | 0 | 0.2 | N/A |  |
| Upper respiratory tract (C30-C33) | 0 | 0.3 | N/A |  |  | 0 | 0.2 | N/A |  |
| Lung/bronchus (C34) | <5 | 1 | 0.98 (0.02-5.51) | 0.632 |  | <1 | 1 | 1.04 (0.03-5.83) | 0.6366 |
| Thymus, heart, mediastinum, pleura, other and ill-defined sites in the respiratory system and intrathoracic (C37-C39) | 0 | 0.1 | n/a |  |  | 0 | 0.1 | n/a |  |
| Bone and cartilage (C40-C41) | <5 | 0.8 | 1.26 (0.33-1.5) | 0.739 |  | 0 | 0.5 | n/a | n/a |
| Malignant skin melanoma and other malignant neoplasms of skin (C43-C44) | 8 | 10.5 | 0.76 (0.33-1.5) | 0.530 |  | 7 | 9.6 | 0.73 (0.29-1.5) | 0.499 |
| Malignant mesothelioma and soft tissue malignancy (C45-C49) | 0 | 1.3 | n/a |  |  | 0 | 1.1 | n/a |  |
| Female genital organs, including ovarian cancer (C51-C52 and C54-C58) | <5 | 4.5 | 0.66 (0.14-1.95) | 0.633 |  | <5 | 4.1 | 0.72 (0.15-2.12) | 0.753 |
| Cervical cancer (C53) | <5 | 3.9 | 1.02 (0.28-2.61) | 0.825 |  | <5 | 3.6 | 1.1 (0.3-2.82) | 0.939 |
| Urinary system (C64-C68) | <5 | 1.4 | 0.73 (0.02-4.09) | 0.911 |  | <5 | 1.3 | 0.79 (0.02-4.41) | 0.838 |
| Eye, brain and other parts of central nervous system (C69-C72) | <2 | 2.5 | 0.79 (0.1-2.88) | 0.991 |  | <1 | 2 | 0.5 (0.001-2.78) | 0.719 |
| Thyroid gland (C73) | <4 | 2.9 | 1.36 (0.37-3.5) | 0.745 |  | <5 | 2.6 | 1.56 (0.42-4.01) | 0.563 |
| Adrenal gland and other endocrine glands (C74-C75) | <5 | 0.4 | 2.5(0.06-14.18) | 0.876 |  | <5 | 0.4 | 2.86 (0.07-16.28) | 0.802 |
| Ill-defined, secondary and unspecified sites (C76-C80) | 16 | 13.9 | 1.15 (0.66-14.18) | 0.665 |  | 12 | 12.5 | 0.96 (0.49-1.67) | 0.992 |
| Hodgkin’s lymphoma (C81) | <5 | 2.2 | 1.38 (0.28-4.05) | 0.829 |  | 0 | 1.8 | n/a |  |
| Non-Hodgkin’s lymphoma (C82-C85) | <5 | 2.7 | 0.37 (0.01-2.05) | 0.458 |  | 0 | 2.1 | n/a |  |
| Multiple myeloma (C90) | 0 |  | n/a |  |  | 0 |  | n/a |  |
| Acute lymphoblastic leukaemia (C91.0) | 0 |  | n/a |  |  | 0 |  | n/a |  |
| Chronic lymphocytic leukaemia (C91.1) | 0 |  | n/a |  |  | 0 |  | n/a |  |
| Acute myeloid leukaemia (C92.0) | 0 |  | n/a |  |  | 0 |  | n/a |  |
| Chronic myeloid leukaemia (C92.1) | 0 |  | n/a |  |  | 0 |  | n/a |  |
